# A Case of Self-salvation in a Determined Chloroquine Suicide Attempt

**DOI:** 10.2478/jccm-2021-0036

**Published:** 2021-11-13

**Authors:** Sylvère Störmann, John Hoppe, Daniela Steinert, Matthias W. Angstwurm

**Affiliations:** 1 Medizinische Klinik und Poliklinik IV, Klinikum der Universität München, Germany

**Keywords:** chloroquine, intoxication, diazepam, suicide attempt

## Abstract

This report concerns a young man who attempted suicide by ingesting a cocktail with a lethal dose of chloroquine phosphate and large amounts of diazepam. On presentation, the patient was drowsy, unresponsive and in cardiogenic shock with severely impaired left ventricular function. Active charcoal and vasopressors were administered, and despite his intoxication with diazepam, a high-dose diazepam treatment was initiated in the hospital. It is concluded that diazepam in the cocktail played a vital role in the survival of this patient. With a rise in numbers, every emergency and intensive care physician should be familiar with chloroquine poisoning.

## Introduction

Suicide is a significant but preventable public health issue. According to the Federal Statistical Office of Germany, approximately 1% of all deaths in 2017 were due to suicide, which is three times as many as road traffic deaths. Most suicides in Germany occur through strangulation (45%). Intoxication with medical drugs is rare and occurred in one out of ten successful suicides, accounting for 0.1% of all deaths in Germany. Unfortunately, data on suicide attempts are scarce and rely primarily on estimation and extrapolation.

The use of chloroquine as a suicidal agent is rare [1], but recently it has gained attention in some European countries due to increased media coverage [2].

The case of a young male patient who attempted suicide using chloroquine tablets in a lethal dose is detailed.

## Case report

A 25-year old male was admitted to the ICU at a university hospital in Munich, Germany, by the emergency medical physician, following an initial diagnosis of suspected ingestion of various drugs as means of suicide. He had made a phone call to his parents approximately five hours before admission to express his love for them. His choice of words and slurred speech alerted the parents. Given a history of suicide attempt four years earlier, they had a strong suspicion that he had just ingested drugs with the intent to commit suicide. The parents immediately called the police, who started an investigation to track down the patient. He was found roughly four hours after the alert in a hotel room nearby the hospital. Emergency staff joined the police forces five minutes later, and the patient arrived in the ICU 40 minutes after being found.

Upon presentation, vital parameters were unsuspicious; heart rate 92/min, blood pressure 111/78 mmHg, breathing frequency 21/min, and blood gas analysis revealed a balanced acid-base metabolism (pH 7.40, pCO2 39.4 mmHg, BE -0.2 mmol/l). The lactate levels (2.2 mmol/l) and normal but mild hypokalaemia (3.3 mmol/l) was recorded. The patient was drowsy, and movements were slow, but he could open his eyes in response to voice. The patient was not able to give adequate responses and uttered indistinct sounds. The Glasgow Coma Scale score was 9 (eye: 3; verbal: 2; motor: 5). His pupils were mid-dilated and responded slowly to light and dark. It was noted that he was a well-developed, well-nourished, and athletic young man. Respiratory excursions were full and symmetrical. Cardiac, respiratory and gastrointestinal auscultation were unremarkable. He had neither abnormal abdominal tympany nor direct or rebound tenderness, rigidity, or guarding. The patient presented with normal skin temperature, and the extremities were not swollen. All pulses were regular. Due to the above mentioned neurological symptoms, an assessment of possible ocular or auricular function defects was not possible. The first responders had found a web page printout with instructions on how to commit suicide alongside a package insert of chloroquine phosphate Avloclor® (Alliance Pharmaceuticals, Chippenham, UK) and an emptied blister of metoclopramide MCP-ratiopharm® (Ratiopharm GmbH, Ulm, Germany) in the hotel room where the patient was initially found.

Therapy was immediately started by administering 50 g active charcoal through a nasogastric tube. We briefly considered lipid emulsion, but as it is not generally thought to be effective in chloroquine poisoning due to the high volume of distribution, we opted against it [3].

An ECG obtained immediately after admission showed broadened QRS complexes of 120 to 130 ms and a prolonged frequency-corrected QT time of 535 ms. Echocardiography performed five minutes after admission revealed cardiomyopathy with highly impaired left ventricular function.

His blood pressure gradually dropped over the next forty-five minutes, suggesting the onset of cardiogenic shock. Vasopressor agents were immediately started. First, epinephrine 0.4 mg/h per intravenous route (Suprarenin®, Cheplapharm Arzneimittel GmbH, Mesekenhagen, Germany) and five minutes later norepinephrine 0.4 mg/h intravenously (Arterenol®, Cheplapharm Arzneimittel GmbH, Mesekenhagen, Germany).

Following a team discussion and considering the generally accepted recommendations, the patient was intubated ninety minutes after admission. Mechanical ventilation was started to allow for a high-dose diazepam infusion with a bolus of 160 mg per intravenous route right after successful intubation followed by 4 mg/kg body weight/24h (Diazepam-® Lipuro, B. Braun Melsungen AG, Melsungen, Germany) [4, 5, 6].

The patient quickly stabilised, and ECG indicated a return to normal parameters within a few hours.

The patient’s parents were contacted two hours after admission to obtain further information on his medical history; the parents also searched his apartment for clues as to the cause of his actions.

It was established from impoundment notices from the customs office that the patient had prepared to commit suicide for at least five months beforehand but was facing difficulties acquiring the necessary drugs. Finally, he had obtained the chloroquine from a British pharmacy acting on behalf of a medical online consultation company registered on an island in the Caribbean Sea (formally belonging to the Netherlands). All of these actions were within European regulations.

The patient had ground several drugs with a porcelain mortar which he had borrowed from his mother. In addition, several emptied blisters of drug packets were found in the patient’s apartment. The total count of drugs amounted to 10 g chloroquine phosphate Avloclor® issued by the afore-mentioned British pharmacy (Alliance Pharmaceuticals, Chippenham, UK), 900 mg diazepam labelled “Diazepam Ratiopharm, 10 mg comprimidos” (Ratiopharm Lda., Carnaxide, Portugal), 25 mg lorazepam labelled “Lorazepam Labesfal 2,5 mg Comprimidos” (Generis® Farmacêutica, S.A., Amadora, Portugal) and 100 mg metoclopramide MCP-ratiopharm® (Ratiopharm GmbH, Ulm, Germany).

On admission, his serum chloroquine levels were 2.300 ng/ml; the therapeutic reference range provided by the toxicology laboratory was 50-200 ng/ml. This slowly dropped to 210 ng/ml on Day 4 post-admission.

Finally, chloroquine levels fell to 48 ng/ml, a therapeutically relevant concentration by Day 8 post-admission.

In order to prevent the rebound phenomenon previously described in a patient after withdrawal of diazepam infusion [7], the dose of diazepam infusion was lowered slowly, following close monitoring of any ECG changes.

After extubation, on Day 7 post-admission, the patient recovered quickly; cardiac output was normal, respiratory and gastrointestinal function were stable, an ophthalmologic assessment was inconspicuous, and there were no signs of neurologic failure.

On gaining responsiveness, the first question he asked was, “How is it possible that I was found?” He admitted preparing the deadly cocktail well in advance and having ingested it with a glass of water in the aforementioned hotel room.

He had no recollection of calling his parents. The patient was transferred to the hospital’s psychiatric clinic for further assessment and treatment two days later.

## Discussion

Chloroquine is an antimalarial agent first synthesised and studied by German researchers in the 1930s [8] and has also been used in rheumatoid arthritis and lupus erythematosus since the 1950s. It has a low margin of safety, and side effects are common. As it is quickly absorbed from the gastrointestinal tract, acute poisoning leads to the sudden onset of severe symptoms [9]. Especially cardiovascular toxicity is common. Blockade of sodium channels decreases cardiac contractility and slows conduction, while blockade of cardiac potassium channels leads to delayed ventricular repolarisation and QT prolongation [6].

Furthermore, arterial vasodilatation occurs frequently and, combined with reduced cardiac inotropy, often leads to cardiogenic shock. Finally, due to its high toxicity and rapid onset of death following ingestion, it was identified as a suicidal agent, the first such reports emerging in the 1960s [10,11].

In the 1980s, the French handbook *Suicide mode d’emploi* (“suicide instruction manual”) describing mechanisms of suicide further popularised this method amongst suicidal persons giving rise to a high number of cases throughout the 1980s and 90s.

Incidentally, a beneficial effect of diazepam in chloroquine intoxication was suspected based on the observation of non-lethal outcomes in cases of combined intoxication, which led to experimental studies in rats and pigs demonstrating a protective effect of diazepam [12,13]. However, few trials have been conducted to assess the value of using diazepam in chloroquine intoxication with mixed results. Riou et al. (1988) reported treating eleven patients who had ingested a dose of chloroquine of more than 5 g with a high-dose diazepam infusion after being admitted to an intensive care unit. Ten of these patients survived.

They compared this group to historical controls where 10 out of 11 cases died. This led to a consensus statement in which routine administration of diazepam in chloroquine intoxications was recommended.

However, this finding was challenged by a retrospective study consisting of 309 patients, of which 163 received diazepam. No difference in mortality was reported, and the study concluded that diazepam’s role in chloroquine intoxication might have been overestimated.

Subsequently, Clemessy et al. (1996) performed the only prospective randomised clinical trial of diazepam versus placebo in moderate chloroquine intoxication. They primarily analysed electrophysiological parameters to clarify the suspected mode of action [14]. Not a single patient in this study died. Therefore, they concluded that diazepam had no effect on positive outcomes and that supportive intensive care is essential in the recovery of chloroquine intoxications. The same group summarised their treatment experience over five years in 167 patients with acute chloroquine poisoning, of which 85% had received diazepam during treatment. The overall outcome was good, and only 14 patients died. Still, those patients had also received diazepam, which suggested the absence of an actual antidotal effect [15]. To this date, no proof of an antidotal effect or lack thereof exists.

Consequently, the consensus recommendations have not been updated, and multiple reports of successful usage of diazepam in chloroquine intoxication have been published. Accordingly, diazepam infusion has remained the de facto standard for treating such cases [16]. Clinically it is noteworthy that several reports have described a tapered sedative effect of diazepam in chloroquine intoxication [17,18], which was also the case in our patient, and additional propofol was necessary to maintain sedation during mechanical ventilation. This suggests a reciprocal effect between chloroquine and diazepam. We suspect the high doses of co-administered diazepam to have played a key role in the survival of our patient.

The toxicological analysis had to be performed in an external laboratory, so we could not effectively guide our treatment by chloroquine levels. The lethal dose of chloroquine for an adult is estimated at 30–50 mg/kg [3]. Mortality correlates with peak blood chloroquine concentration [15], and various cut-offs have been proposed, but there are some concerns in the measurement of chloroquine levels. Firstly, there is evidence for interindividual variation due to differences in metabolism. Secondly, chloroquine has an enormous volume of distribution and is rapidly distributed into the various body compartments escaping measurement in blood samples [19]. Chloroquine levels from different sources should be compared with caution as type (whole blood vs serum vs plasma), storage, and handling of samples largely influence the measure [20,21]. Therefore, comparability within a single centre and laboratory is feasible, but not between different centres, laboratories, and especially sample types. Only serum samples were available from the current patient, which effectively measured the least possible concentration of chloroquine not yet distributed into cells and tissue.

Peak chloroquine concentration occurs within 1.5 to 3 hours after ingestion [22]. Our patient’s peak concentration was measured in the sample at admission just after administration of active charcoal (approximately 5 hours after ingestion) with 2.3 mg/l (corresponding to 7.2 μmol/l). This may seem small compared to published levels, but those are derived from whole blood or plasma and sampled earlier; that is when higher levels are expected. Of course, one could argue that the patient did not ingest as much chloroquine as suspected, but the patient confirmed the intake of the amount mentioned above of chloroquine and diazepam tablets. As stated in the case description, the patient had prepared for this suicide attempt for at least five months. Afflicted with anhedonia and feeling of numbness and callousness, as well as delusion and derealisation, he had attempted suicide by jumping off a hotel room a few months earlier, which had resulted in severe injury, including incomplete paraplegia. After successful physical recovery, the patient still complained of emptiness inside that made him feel numb. Still, he managed to keep his inner turmoil hidden. Even though he did not expect first responders to find him so soon, he had anticipated the possibility of being found and, for this case, created a living will refusing resuscitation and placed it clearly visible in the hotel room. Realising this had failed, he was furious that his living will had been ignored and announced wanting to take legal action against the first-aiders.

In summary, there is no doubt about the patient’s resolution to commit suicide.

Suicide with chloroquine is generally rare but seems to increase. The United States’ National Poison Data System annually reports toxicological fatalities since 1983 [23]. Intentional suicide involving (hydroxy-) chloroquine was registered in only 4 cases during the 1980s, five times in the 1990s, slightly increasing to 7 instances in the 2000s and already counting 15 victims from 2010 on [24]. Similarly, official data on mortality collected by the Federal Statistical Office of Germany counts a total of 29 deaths related to poisoning with antimalarial agents in 20 years (1998-2017). However, 20 of those occurred within the last seven years, indicating a rise in numbers. Just as the publication of the instructional handbook *Suicide mode d’emploi* in the 1980s led to more chloroquine-related suicides, Navarro Escayola and colleagues consider accessibility to instructional websites that may include recommendations for chloroquine suicide cocktails to be responsible for such an increase. In addition, chloroquine is easily obtained, and the growth of the online pharmacy sector may facilitate the procurement of such drugs even more.

Furthermore, recent media coverage on the potential use in SARS-CoV-2 infection and its toxicity may raise awareness of chloroquine, particularly in the general population. Therefore, there is reason to expect a further rise in potentially fatal chloroquine poisonings despite its rarity. Therefore, every emergency and intensive care physician should be aware of this form of intoxication.

In conclusion, the de facto standard of early intubation and diazepam infusion in high-dose chloroquine intoxication is effective. However, in our case, co-administration of high doses of diazepam by the patient itself may have contributed to the positive outcome.
